# “Test and not treat” for onchocerciasis control in a *Loa loa* endemic area

**DOI:** 10.1056/NEJMoa1705026

**Published:** 2017-11-08

**Authors:** Joseph Kamgno, Sébastien D. Pion, Cédric B. Chesnais, Matthew H. Bakalar, Michael V. D'Ambrosio, Charles D. Mackenzie, Hugues C. Nana-Djeunga, Raceline Gounoue-Kamkumo, Guy-Roger Njitchouang, Philippe Nwane, Jules B. Tchatchueng-Mbouga, Samuel Wanji, Wilma A. Stolk, Daniel A. Fletcher, Amy D. Klion, Thomas B. Nutman, Michel Boussinesq

**Affiliations:** Centre for Research on Filariasis and other Tropical Diseases, Yaounde Cameroon; Faculty of Medicine and Biomedical Sciences, University of Yaounde I, Yaounde Cameroon; IRD UMI 233-INSERM U1175-Montpellier University, Montpellier, France; IRD UMI 233-INSERM U1175-Montpellier University, Montpellier, France; Department of Bioengineering & Biophysics Program, University of California - Berkeley, Berkeley, CA 94720, US; Department of Bioengineering & Biophysics Program, University of California - Berkeley, Berkeley, CA 94720, USA; Department of Pathobiology and Diagnostic Investigation, Michigan State University, East Lansing, USA; Centre for Research on Filariasis and other Tropical Diseases, Yaounde Cameroon; Centre for Research on Filariasis and other Tropical Diseases, Yaounde Cameroon; Centre for Research on Filariasis and other Tropical Diseases, Yaounde Cameroon; Centre for Research on Filariasis and other Tropical Diseases, Yaounde Cameroon; Centre for Research on Filariasis and other Tropical Diseases, Yaounde Cameroon; Department of Public Health, ErasmusMC, University Medical Center, Rotterdam, the Netherlands; Department of Bioengineering & Biophysics Program, University of California - Berkeley, Berkeley, CA 94720, USA; Chan Zuckerberg Biohub, San Francisco, CA 94158, USA; Laboratory of Parasitic Diseases, National Institute of Allergy and Infectious Diseases, Bethesda, MD 20892, USA; Laboratory of Parasitic Diseases, National Institute of Allergy and Infectious Diseases, Bethesda, MD 20892, USA; IRD UMI 233-INSERM U1175-Montpellier University, Montpellier, France

## Abstract

**Background:**

Implementation of ivermectin-based community treatment for onchocerciasis or lymphatic filariasis elimination has been delayed in Central Africa because of severe adverse events (SAEs), including death, in people with high levels of circulating *Loa loa* microfilariae (mf). LoaScope, a rapid field-friendly diagnostic tool to quantify *L. loa* mf in peripheral blood, permits point-of-care identification of individuals “at risk” for SAEs.

**Methods:**

A “Test and not Treat” (TaNT) strategy was used to implement ivermectin treatment in the Okola health district in Cameroon, where ivermectin distribution was halted in 1999 after the occurrence of fatal *Loa*-related SAEs. The LoaScope was used to identify and exclude individuals with >20,000 mf per milliliter of blood (at-risk for SAEs) from ivermectin treatment. Active surveillance for post-treatment adverse events (AEs) was conducted daily for 7 days.

**Results:**

Between August and October 2015, 16,259 (71.1%) individuals >=5 years of age were tested out of a target population of ~22,800. Among the ivermectin-eligible population, 15,522 (95.5%) received ivermectin; 340 (2.1%) were excluded from ivermectin treatment because of a *L. loa* density above the risk-threshold and 397 (2.4%) were excluded for pregnancy or illness. No SAEs were observed. Non-severe AEs were recorded in 934 individuals, most (67%) of whom had no detectable *L. loa* mf.

**Conclusions:**

The LoaScope-based TaNT strategy permitted safe re-implementation of community-wide ivermectin distribution in a heretofore ‘off limits’ health district in Cameroon and is an extremely promising and practical approach for large-scale ivermectin treatment for lymphatic filariasis and onchocerciasis elimination in *Loa loa*-endemic areas.

## Introduction

Mass drug administration (MDA) with ivermectin-containing regimens is the main strategy for elimination of lymphatic filariasis and onchocerciasis. Although generally safe, ivermectin distribution has led to severe adverse events (SAEs) in central African countries. More than 500 cases of characteristic post-ivermectin encephalopathy^1^ including ~60 fatal case, occurred during MDA and have therefore been reported to the Mectizan Donation Program since 1990. Of note, these neurologic SAEs have occurred exclusively in individuals with peripheral blood *Loa loa* microfilarial densities >30,000 microfilariae (mf) per milliliter,^1^ ^2^ and are presumed to be related to eosinophil-mediated inflammation around dying mf and/or micro-embolization with subsequent loss of central nervous system vascular integrity.

Current WHO guidelines allow ivermectin-based MDA to be implemented in areas where onchocerciasis is meso- or hyperendemic because the potential benefits of MDA are felt to outweigh the risk of ivermectin-associated SAEs, although enhanced surveillance for adverse events (AEs) is required. However, areas endemic for loiasis and hypo-endemic for onchocerciasis are spread throughout Central Africa,^3^ and remain a serious problem. For these areas, a “Test and (not) Treat” (TaNT) strategy has been proposed, wherein individuals with high *L. loa* mf loads (at risk for SAEs) are excluded from ivermectin treatment and the remaining population (typically >95%) can be treated safely.

Successful implementation of the TaNT strategy requires a rapid, point-of-contact, fieldfriendly and highly accurate method to quantify *L. loa* mf. To this end, a mobile phone-based videomicroscope – the LoaScope (previously CellScope Loa) – was developed.^4^ The LoaScope automatically counts *L. loa* mf in peripheral blood collected in disposable rectangular capillaries without the need for sample processing using a smartphone coupled to a simple optical device (Figure S1 and Movie S1).^4^

To advance *O. volvulus* elimination in *L. Loa* co-endemic countries in Central Africa, we tested the feasibility of this TaNT strategy in the Okola health district in Cameroon, where ivermectin distribution was halted in 1999 after the occurrence of *Loa*-related encephalopathy. As such, the TaNT strategy allowed the safe reintroduction of ivermectin in all of the communities in this health district without provoking a single SAE.

## METHODS

### Study site

The Okola health district (Figure S2) includes 11 health areas where, in 1999 MDA was halted by the Ministry of Public Health following 23 cases of encephalopathy that occured during the first treatment campaign. MDA only resumed in 5/11 areas deemed hyper- or mesoendemic (>20% onchocercal nodule prevalence in adult males). In 2013, nodule surveys in the 6 excluded health areas had prevalences of 6% to 40% consistent with hypo- or meso-endemic onchocerciasis.^5^ The entire Okola health district is known to be highly endemic for *L. loa*.^6^ ^7^

### Study design

The TaNT strategy was implemented in the 92 villages of the 6 health areas untreated since 1999 (Figure S2). The timeline of the TaNT project is depicted in Figure S3 and the process is described in the Supplementary Methods. All individuals aged >=5 years were invited to participate.

The TaNT process consisted of registration of consenting (or assenting) individuals >=5 years of age, LoaScope quantification of *L. loa* microfilarial density, treatment of eligible individuals with ivermectin (150μg/kg) and surveillance for AEs. Non-pregnant subjects excluded from ivermectin distribution because of high *L. loa* mf counts were given albendazole (400 mg) for intestinal deworming. Self-declared pregnant women were not treated with ivermectin or albendazole, but were offered iron and folic acid tablets. Each participant was given a card (Figure S4) with their *L. loa* mf count, the treatment received and a contact phone number for questions and/or reporting of AEs.

### Quantification of *L. loa* microfilaremia

The use of the LoaScope and its performance have been described previously.^4^ A threshold of 26,000 mf per milliliter was initially selected for ivermectin treatment, based on the lower 95% confidence interval around the 30,000 mf per milliliter threshold below which no neurologic SAEs were observed in prior studies^8-10^ and the calculated false negativity rate of 1 in 10 million (<0.00001%).^4^ Two weeks after the study start, a case of conjunctival hemorrhage, similar to those described previously,^11^ occurred in a subject with a LoaScope *L. loa* mf count of 24,599 mf per milliliter. For potential safety reasons, the exclusion threshold of the LoaScope was decreased to 20,000 mf per milliliter for the remainder of the trial.

Calibrated (50 μL) thick smears were performed as a backup for samples unable to be analyzed with the LoaScope, to identify and quantify *Mansonella perstans* mf, and to corroborate the accuracy of the LoaScope. Smears were read by 2 different microscopists blinded to the LoaScope results. Dried blood spots collected on filter paper were archived and stored at -80°C.

### Assessment of exposure to onchocerciasis

Ov16 IgG4 antibodies positivity from eluted single blood spots (10 μl equivalent) was determined the SD Bioline Onchocerciasis IgG4 Rapid Test.^13,14^

Results were read and recorded at 24 hours.

### Monitoring of post-treatment adverse reactions

Monitoring for AEs was performed by two surveillance teams, each composed of a physician and a driver, with the assistance of selected community members (pre-Community Drug Distributors (pre-CDDs)) and local nurses. The surveillance teams visited each village on days 1, 2, 3 and 6 post-treatment, examined all individuals complaining of AEs and provided symptomatic treatment if indicated. In addition, the team toured the entire community by car to identify additional individuals with AEs. All AEs were recorded using a standardized form (Figure S5). A Karnofsky performance score index was assigned to each patient examined. Clinical management was based on reference guidelines.^15^

### Statistical analysis

Medians and interquartile ranges were used as measurements of central tendency. Associations between individual factors (gender, age, *L. Loa* mf density (assessed by LoaScope), presence of Ov16 IgG4, presence of *M. perstans* mf (assessed by calibrated blood smear microscopy) and the occurrence of AEs were assessed using multivariable logistic regression.

The logistic regression coefficients were used to calculate population attributable fractions.^16^ The calibrated thick smear was used as the reference test for assessment of the specificity and negative predictive value of the LoaScope.

### Ethical agreement

This study was authorized by the National Ethics Committee of Cameroon (ethical clearance n° 2013/11/370/L/CNERSH/SP) and approved by the Division of Operational Research at the Ministry of Health (Administrative authorization n° D30- 571/L/MINSANTE/SG/DROS/CRSPE/BBM). All volunteers provided written signed consent (or parental consent in the case of minors) before undergoing blood sampling and again before receiving treatment.

### Authors’ contributions to the study

Authors’ contributions to this study are as follows: JK, SDP, CDM, ADK, TBN and MB designed the study; JK, SDP, CBC, MHB, MVDA CDM, HCND, RGK, GRN, PN, JBTM, SW, DAF, ADK, TBN and MB gathered the data; SDP and CBC analyzed the data; TNB and MB vouch for the data and the analysis; MHB, MVDA, DAF provided diagnostic technology development and support; JK, SDP, CDM, HCND, WAS, DAF, ADK, TBN and MB wrote the paper; and JK, SDP, CDM, DAF, ADK, TBN and MB decided to publish the paper. SDP wrote the first draft of the manuscript.

## RESULTS

### Population Characteristics

**Figure 1.**
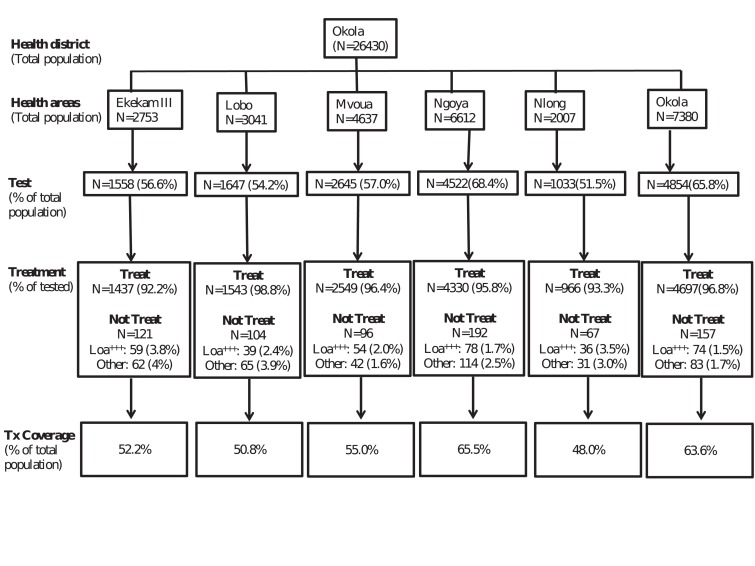
Flowchart of the population examined for L. loa microfilaremia and treated with ivermectin in the six health areas of the Okola district. Loa+++ indicates the number of individuals identified as at risk of post-ivermectin severe adverse events and excluded from ivermectin treatment.

A total of 16,259 individuals were examined during the TaNT process (Figure 1). The median age of the examined populations ranged from 17 to 26 years in the different health areas and the sex distribution was relatively equal (48% male). The prevalence of Ov16 IgG4 antibody in the six health areas varied from 15.3% to 29.9%. The prevalence of *L. loa* microfilaremia varied from 15.3% to 22.8%, and the proportion of individuals with more than 20,000 *Loa* mf per milliliter as determined by LoaScope ranged from 1.3% in the Ngoya health area to 2.4% in the Nlong and Ekekam III health areas (Table 1, Table S2).

**Table 1.** Demographics, onchocerciasis prevalence and *L. loa* microfilaremia levels in the population of the six health areas of the Okola district (Cameroon)

.	Census	Median age (IQR)	M/F	*O. volvulus* antibody positivity (%)	*L. loa* microfilaremia (%)			
					Prevalence	< 8000 mf per milliliter	8-20,000 mf per milliliter	> 20,000 mf per milliliter
Ekekam III	2753	26 (13-49)	0.51	18.8	22.8	91.7	5.9	2.4
Lobo	3041	23 (12-45)	0.50	29.9	21.3	93.1	5.3	1.6
Mvoua	4637	17 (10-43)	0.49	20.2	18.9	94.4	3.7	1.9
Ngoya	6612	17 (10-38)	0.47	15.3	15.3	95.4	3.3	1.3
Nlong	2007	20 (12-50)	0.51	23.8	20.3	94.5	3.1	2.4
Okola	7380	18 (11-38)	0.58	27	16.1	95.1	3.5	1.4
Total	26430	18 (11-42)	0.48	22.4	17.8	94.5	3.9	1.6

**Table 2.** Adverse events recorded during the post-treatment surveillance process.

Adverse Events	No of adverse events	No. with *Loa* mf (%)	No. with no *Loa* mf (%)	P value
Pruritus	564	188 (33.3)	376 (66.7)	<0.001
Asthenia	389	171 (44)	218 (56)	0.002
Headache	326	149 (45.7)	177 (54.3)	0.14
Rash	274	52 (19)	222 (81)	<0.001
Back Pain	257	128 (49.8)	129 (50.2)	0.97
Arthralgias	235	124 (52.8)	111 (47.2)	0.39
Edema	125	21 (16.8)	104 (83.2)	<0.001
Myalgia	115	51 (44.4)	64 (55.6)	0.20
Vertigo	106	48 (45.3)	58 (54.7)	0.35
Anorexia	89	42 (47.2)	47 (52.8)	0.57
Abdominal pain	67	19 (28.4)	48 (71.6)	<0.001
Blurred vision	66	27 (40.9)	39 (59)	0.15
Difficulty ambulating	58	27 (46.6)	31 (53.4)	0.65
Diarrhea	46	18 (39.1)	28 (60.9)	0.14
Difficulty in getting upright	37	20 (54)	17 (46)	0.63
Lymphadenopathy	23	8 (34.8)	15 (65.2)	0.17
Conjunctival hemorrhage	20	14 (68.4)	6 (31.6)	0.14
Conjunctival itching	13	7 (53.9)	6 (46.2)	0.77
Tinnitus	6	4 (66.7)	2 (33.3)	Not tested
Temporary hearing loss	2	0 (0)	2 (100)	Not tested
Total	2818	1118 (39.7)	1702 (60.4)	<0.001

### Test and Not Treat

Between 50 and 100 participants were typically examined per village per day. The mean time from finger prick to LoaScope result was 2-3 minutes. The LoaScope results were immediately available for 16,099/16,259 individuals (99%) and were delayed for 160 individuals (1%) because of technical problems requiring determination of the mf count by calibrated thick smear. Ivermectin was administered to 15,522 individuals (95.5%) with mf levels below the established threshold.

Seven hundred and thirty seven (4.5%) subjects were excluded from ivermectin therapy. Of these, 340 (2.1%) were excluded because of a *L. loa* density above the risk-threshold, 228 (1.4%) because of poor health (signs or symptoms consistent with a serious acute or chronic concomitant illness) or inebriation, and 169 (1%) because of pregnancy or breastfeeding. The proportion of excluded individuals per village varied from 0% to 15.1% (Figure S6). All excluded individuals (except pregnant women), were treated with albendazole (400 mg). The median treatment coverage in the district was 55% of the total population (interquartile range between villages: 42.9–64.1%), and 64% of the targeted population.

The prevalence of *O. volvulus*-specific antibody (Ov16 IgG4) was 22.0% in individuals who received ivermectin, 25.4% in those excluded for pregnancy or illness, and 33.5% in those with a *L. loa* density above the risk-threshold. Thus, individuals who were not treated because of *Loa* microfilaremia and who were potentially infected with O. volvulus represented only 0.7% of the examined population.

### Frequency and types of AEs

Among the 15,522 individuals treated with ivermectin, 934 (6%) had documented AEs. The incidence of AEs decreased slightly from 6.6% (464/7,065) to 5.6% (470/8,457) (p<0.0001) after reducing the exclusion threshold from 26,000 to 20,000 mf per milliliter. Dermatologic manifestations were most common, followed by systemic and rheumatologic manifestations (Table 2). Eight hundred and sixty-nine people (93%) had a Karnofsky score of 90, and 65 (7%) had a score of 80. All AEs resolved within one week without treatment or with basic supportive therapy (anti-histamines, non-steroidal anti-inflammatory drugs, or acetaminophen).

**Figure 2.**
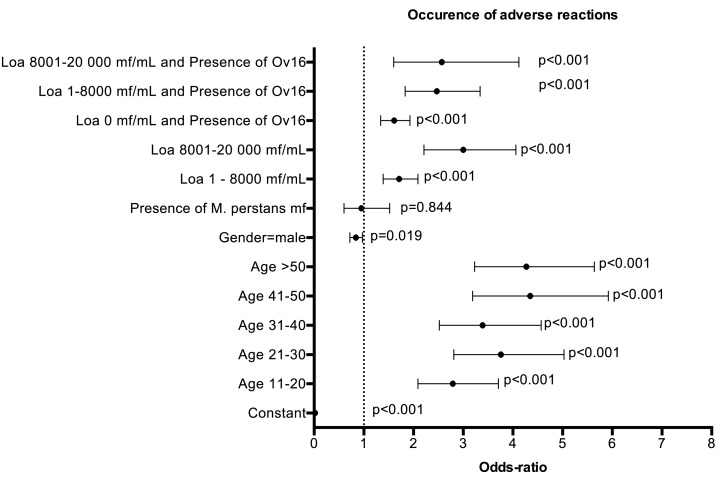
Results of multivariate logistic regression of occurrence of post-ivermectin adverse events in relation to individual factors Dots with error bars represent odds-ratios (OR) and 95% confidence intervals. The dotted line (OR=1) 1 represents an absence of association.

Both *L. loa* microfilaremia and the presence of Ov16-specific IgG4 were assessed in 888 of the 934 individuals who developed an AE. Among these, 43.2% had neither *L. loa* mf nor Ov16 IgG4, 22.3% had only *L. loa* mf, 23.9% had only Ov16 IgG4, and 10.6% had both *L. loa* mf and Ov16 IgG4. Multivariable regression indicates that AEs were significantly more frequent in older individuals, females, and individuals with either *L. loa* mf or Ov16 IgG4 (Figure 2). The risk of AEs associated with presence of Ov16 IgG4 was similar to that associated with harboring 1-8000 *Loa* mf per milliliter (Odds ratio (OR)=1.61 and 1.71, respectively) and was about half the risk associated with harboring 8000-20,000 *Loa* mf per milliliter (OR=3.00). The risk of AEs associated with both *L. loa* microfilaremia and O. volvulus IgG4 was similarly increased in persons harboring 1-8000 *L. loa* mf per milliliter (OR=2.47) and in those harboring 8000-20,000 *L. loa* mf per milliliter. Population attributable fractions of AEs for *L. loa* mf density of 1-8000 mf per milliliter, 8000-20,000 mf per milliliter and Ov16 IgG4 were 8.0%, 8.3% and 12.2%, respectively.

### Agreement between LoaScope and calibrated blood smear microscopy

**Figure 3.**
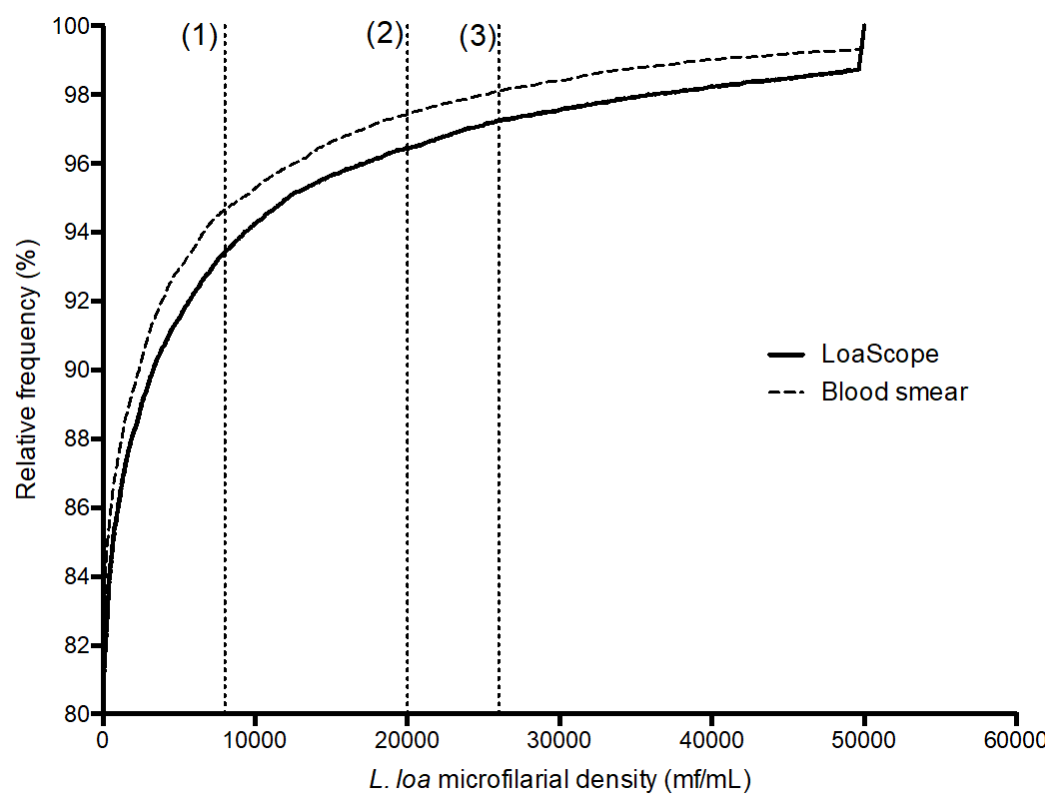
Cumulative frequency distribution of *L. loa* microfilarial density in the population with tails of distribution censored for density above 50,000 mf per milliliter (mL). Dotted vertical lines (1), (2) and (3) correspond to the 8,000 (1), 20, 000 (2) and 26,000 )3) *Loa* mf/ml cutoffs used to determine treatment exclusion thresholds (2 and 3) and information relevant to the increased likelihood of adverse events (1) provided to each participant.

Figure 3 shows that the distributions of *L. loa* mf density in the population using the LoaScope and thick smear microscopy were similar. The specificity and negative predictive values of the LoaScope to identify individuals with mf counts below 20,000 mf per milliliter (as assessed by microscopy) were 99.7% (95% confidence interval: 99.6 – 99.8) and 99.7% (99.6 – 99.7), respectively.

## Discussion

Extension of ivermectin-based MDA to areas hypoendemic for onchocerciasis and coendemic for loiasis remains a significant obstacle to the success of onchocerciasis elimination programs in Africa. In the current study, a LoaScope-based TaNT strategy was used to safely treat more than 15,000 individuals with ivermectin in such an area. Although there was initial reticence to participate in some villages because of the memory of the SAEs (including deaths) that occurred in 1999, 16,259 of the 22,842 individuals aged >= 5 years old recorded during the initial census (71.1%) participated in the TaNT campaign. This suggests that TaNT is an acceptable strategy even in populations with a history of previous ivermectin-related SAEs. Though not formally assessed, it is likely that fear of SAEs was the main reason for non-participation.

During the first MDA campaign conducted in 1999 in Okola, 23 cases of neurological SAEs, including three fatalities, were recorded among the 6,000 individuals who received ivermectin before MDA was stopped.^17^ The incidences of post-ivermectin neurological SAEs and deaths were therefore 38/10,000 and 5/10,000, respectively. Extrapolating these data to the population enrolled in the present study, a minimum of 62 cases of neurological SAEs and 8 deaths were theoretically prevented by TaNT.

Although some individuals (6%) complained of ivermectin-associated AEs during the TaNT campaign, the proportion was lower than that typically observed after ivermectin MDA for onchocerciasis in areas not endemic for loiasis: 13.1% in south-east Nigeria,^18^ 12% and 20% in northern Cameroon,^19^ and 21.4% in eastern Sudan.^20^ It was also much lower than the 26.3% recorded in a neighboring Loa-endemic area of central Cameroon.^2^ The most likely explanation for the lower frequency of AEs recorded in the Okola district is that onchocerciasis is hypo- and mesoendemic in this area.

The LoaScope operators underwent a 1-hour training session 2 weeks before the field operations. This training was sufficient for the entire study, and the teams noted the ease of use and reliability of the device despite daily use and demanding field conditions. Because *L. loa* mf are diurnally periodic,^21^ LoaScope examinations (and treatment) started at 10 am and ended at 4 pm. During this TaNT campaign, up to 162 individuals were examined per village per day.

Whereas the present study clearly shows that the TaNT procedure is safe and feasible at a district level, moving TaNT from the operational research arena to Central African-wide implementation will depend on a number of factors, including greater reliance on local personnel for the census and post-treatment surveillance and smaller teams (a “tester” and a “treater”) for the TaNT process itself. If only a single TaNT round were needed, this would have a major impact on the applicability of this approach at a larger scale. Since ivermectin has marked microfilaricidal and probable embryostatic activity against *L. loa*, marked and sustained reduction in *L. loa* mf density is expected for one year after the TaNT campaign. In fact, in a neighboring district, the average reduction in *L. loa* microfilarial load was >74% one year after a single dose of ivermectin, and no individual with a pre-treatment density <30,000 mf per milliliter had a count above this level after 12 months.^22^ Thus, it seems likely that a single community-wide round of TaNT will be necessary, with individual testing in subsequent years restricted to previously ivermectin-untreated individuals. This hypothesis, as well as operationality, performance and cost of a TaNT conducted by 3-member teams will be assessed in late 2017.

Given the low percentage (2.4%) of the total population excluded from ivermectin treatment and the proposed implementation of TaNT in areas hypo- and meso-endemic for onchocerciasis, it is unlikely that excluded individuals will be a significant reservoir of *O. volvulus* microfilariae at the community level. Nevertheless, some excluded individuals are likely to be infected with *O. volvulus* and, for ethical reasons, should be treated with effective and safe drug regimens, particularly in the setting of clinical manifestations of onchocerciasis. Although a 4-6-week course of doxycycline, a regimen known to be macrofilaricidal for *O. volvulus*^23^ but not *L. loa*^24^, is impractical at the community level, it could be used safely in this context.

In summary, this TaNT strategy based on a novel and scalable point-of-contact tool that allows rapid identification (and exclusion from ivermectin-based treatment) of individuals at risk of *Loa*-related SAEs has enabled district-level community treatment of onchocerciasis. Though this TaNT strategy was motivated by the need to tackle hypoendemic onchocerciasis in Central Africa, it could also be considered for other foci co-endemic for onchocerciasis and loiasis. As many (but not all) meso-hyperendemic areas are already covered by CDTI, TaNT would target ivermectinnaïve individuals and systematic non-compliers.

## Supplementary Material

Supplementary Appendix
