# Supplementary material for: “Test and not treat” for onchocerciasis control in a Loa loa endemic area
Source: N Engl J Med. Author manuscript; Available in PMC 2017 Nov 8. (PMC5629452; doi:10.1056/NEJMoa1705026)
Supplement: Supplementary Appendix [file NEJMoa1705026_Pion_SuppApp.pdf]

Supplementary Appendix

This appendix has been provided by the authors to give readers additional information about their work.

Supplement to: Kamgno et al. **"Test and not treat" for onchocerciasis control in a *Loa loa* endemic area**

Supplementary Appendix, Table of Contents for Supplement Materials

1) Table S1. Individuals examined and treated and number of AEs per village by health area
2) Figure S1. Principle of use and output of LoaScope
3) Figure S2. Location of the 92 villages included in the TaNT survey with Ov16 IgG4 prevalence
4) Figure S3. Timeline of the Test and treat strategy
5) Figure S4. Individual card given to all participants
6) Figure S5. Standardized form to record all adverse events following ivermectin treatment
7) Figure S6. Number of excluded individuals in each village, classified by cause of exclusion
8) Movie S1. Link to a movie of the process from introduction of the capillary to the result
9) Supplementary Methods

Table S1. Individuals examined and treated and number of AEs per village by health area

Health area	Village	No. Exam	Loa microfilaremia (%)	Ov16 (%)	No. AEs
Ekekam III	Bidzinguele	23	30.4	21.7	0
Ekekam III	Ekekam III	138	27.5	18.5	23
Ekekam III	Essakoss	33	21.2	15.6	1
Ekekam III	Eyang	104	22.1	19.4	6
Ekekam III	Ngoas	132	27.3	26.6	25
Ekekam III	Nkolbiyen	66	25.8	14.1	0
Ekekam III	Nkolbot	44	27.3	9.1	2
Ekekam III	Nkolfoundi	72	27.8	38.6	10
Ekekam III	Nkolmelen	21	33.3	23.8	5
Ekekam III	Nkolmeyang	132	20.5	18.0	9
Ekekam III	Nkolomang I	29	20.7	10.3	2
Ekekam III	Nkolomang II	39	25.6	17.9	0
Ekekam III	Nkonmesse	110	26.4	22.7	6
Ekekam III	Otou Ngono	28	14.3	22.2	1
Ekekam III	Ozasingui	56	33.9	32.1	7
Ekekam III	Ozom I	237	8.0	12.5	2
Ekekam III	Ozom II	185	25.9	14.0	13
Ekekam III	Ozom III	53	30.2	13.7	10
Ekekam III	Qbam	56	17.9	18.2	10
Lobo	Akok	117	15.4	33.3	4
Lobo	Elig Abode	99	19.2	35.1	18
Lobo	Koudi	172	30.2	28.8	6
Lobo	Lobo	406	15.8	30.7	31
Lobo	Menguek I	88	21.6	26.2	10
Lobo	Menguek II	79	24.1	28.2	9
Lobo	Menvoa	65	29.2	45.3	10
Lobo	Nkongkadak	114	24.6	43.9	6
Lobo	Ovang	136	15.4	20.0	15
Lobo	Tikong	123	28.5	26.0	6
Lobo	Tsek	273	22.3	25.1	11
Mvoua	Bilono I	190	18.9	21.1	17
Mvoua	Bilono II	189	22.2	17.0	10
Mvoua	Ekabita Tom	212	18.4	12.6	18
Mvoua	Louma	393	19.3	24.8	46
Mvoua	Mvoua	463	20.7	23.8	44
Mvoua	Nkol Bega	317	15.5	11.9	7
Mvoua	Ntsama	308	16.6	23.9	31
Mvoua	Oban I	143	25.2	21.7	15
Mvoua	Okoukouda	212	20.3	17.7	7
Mvoua	Oyama	219	15.1	21.3	25

Table S1 (Continued)

Health area	Village	No. Exam	Loa microfilaremia (%)	Ov16 (%)	No. AEs
Ngoya	Beyidzolo	326	13.5	16.2	2
Ngoya	Ebot	112	30.4	16.7	4
Ngoya	Essong Aboudi	157	18.5	11.7	2
Ngoya	Etoud	146	22.6	23.4	8
Ngoya	Leboudi	590	7.7	22.3	13
Ngoya	Metak	158	24.1	16.3	2
Ngoya	Minsole	151	20.5	11.3	17
Ngoya	Mintotomo	242	15.7	16.2	5
Ngoya	Ngoya I	496	11.7	13.9	10
Ngoya	Ngoya II	265	14.7	10.2	8
Ngoya	Nkloessong	281	14.9	16.1	8
Ngoya	Nklondom	121	25.6	18.2	1
Ngoya	Nkolenyeng	81	13.6	9.9	1
Ngoya	Nkolngon	319	13.2	12.8	11
Ngoya	Nkong	119	18.5	22.4	3
Ngoya	Nouma	380	20.4	17.1	13
Ngoya	Yagassi	288	18.8	13.0	27
Ngoya	Zamengoue	290	7.3	10.0	16
Nlong	Akak	14	35.7	42.9	0
Nlong	Ebenga	25	16.0	40.0	0
Nlong	Ekoumtik	221	18.6	32.9	12
Nlong	Elig Menyenke	87	18.4	14.6	1
Nlong	Elig Vondo	91	18.7	23.6	4
Nlong	Kelle	69	21.7	32.4	1
Nlong	Mbama	91	16.5	11.1	4
Nlong	Ngondzie	40	17.5	22.5	4
Nlong	Ngoulmakong	90	21.1	14.4	2
Nlong	Nklongock	95	30.5	28.3	2
Nlong	Nkolbeyegle	53	13.2	17.0	0
Nlong	Nkolget	40	27.5	25.0	10
Nlong	Nlong	88	20.5	17.9	2
Okola	Leboth	323	17.6	25.1	30
Okola	Ledom I	292	13.4	39.6	15
Okola	Ledom II	250	17.2	33.5	13
Okola	Legom	394	11.7	28.5	18
Okola	Lekie Assi	296	15.2	25.4	12
Okola	Nkoldjobe	311	22.5	23.9	12
Okola	Nkolessong	137	16.8	30.9	0
Okola	Nkolfeb	147	21.8	17.9	6
Okola	Nkolmekouma	126	17.5	27.6	10
Okola	Nkolnyada	337	22.6	23.0	17
Okola	Nkolodou	153	21.7	30.7	5
Okola	Obak	346	22.5	22.6	23
Okola	Oban II	293	21.5	33.6	26
Okola	Okola – Centre	345	6.1	22.2	18

Okola	Okola – Camp Sap	142	4.9	29.3	3
Okola	Okola – Haoussa	464	11.4	24.0	23
Okola	Okola – Nylon	238	14.3	23.6	15
Okola	Okola – Zanzibar	260	15.0	30.7	19

Figure S1. Principle of use and outputs of LoaScope.

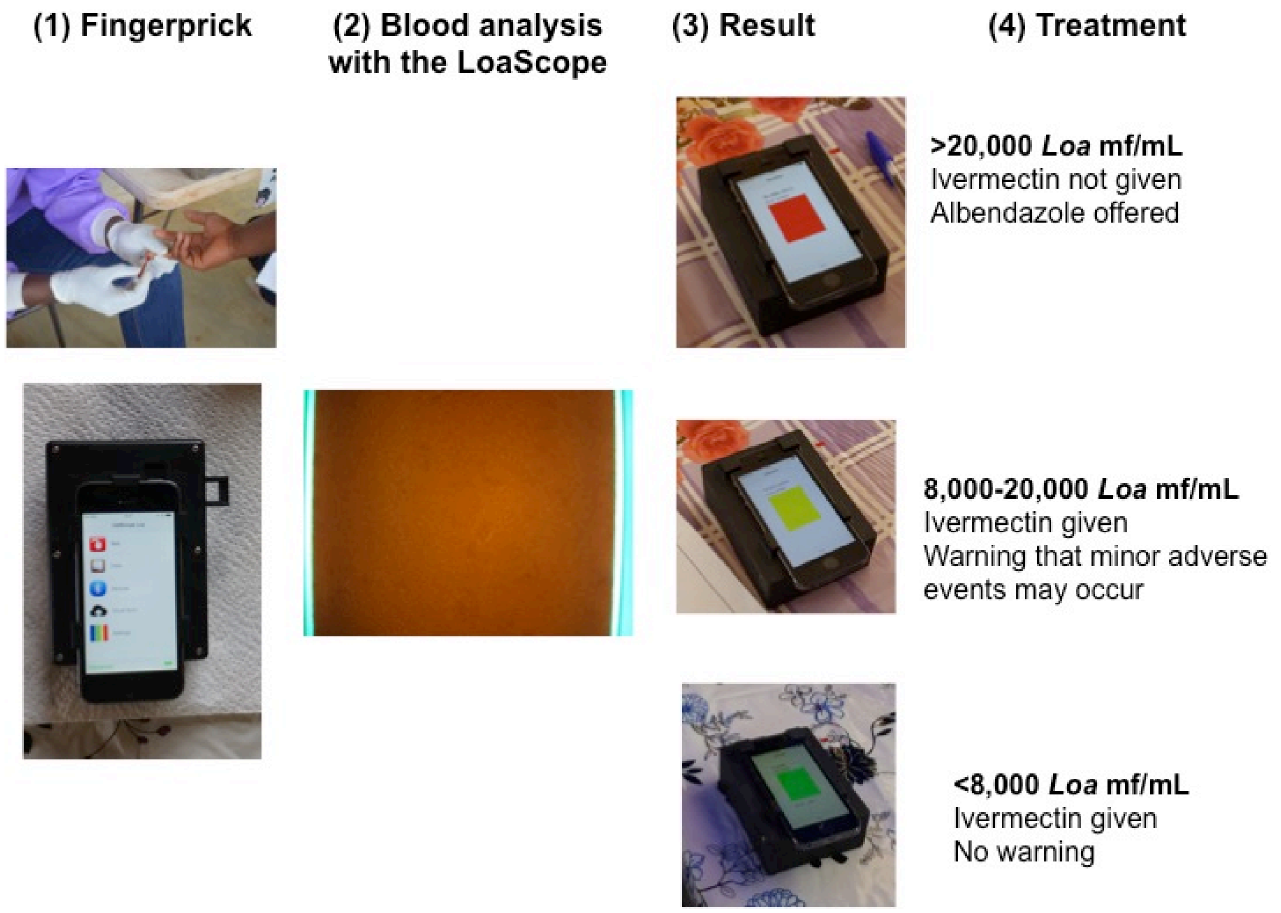

Figure S2. Location of the 92 villages included in the TaNT survey with Ov16 prevalence.

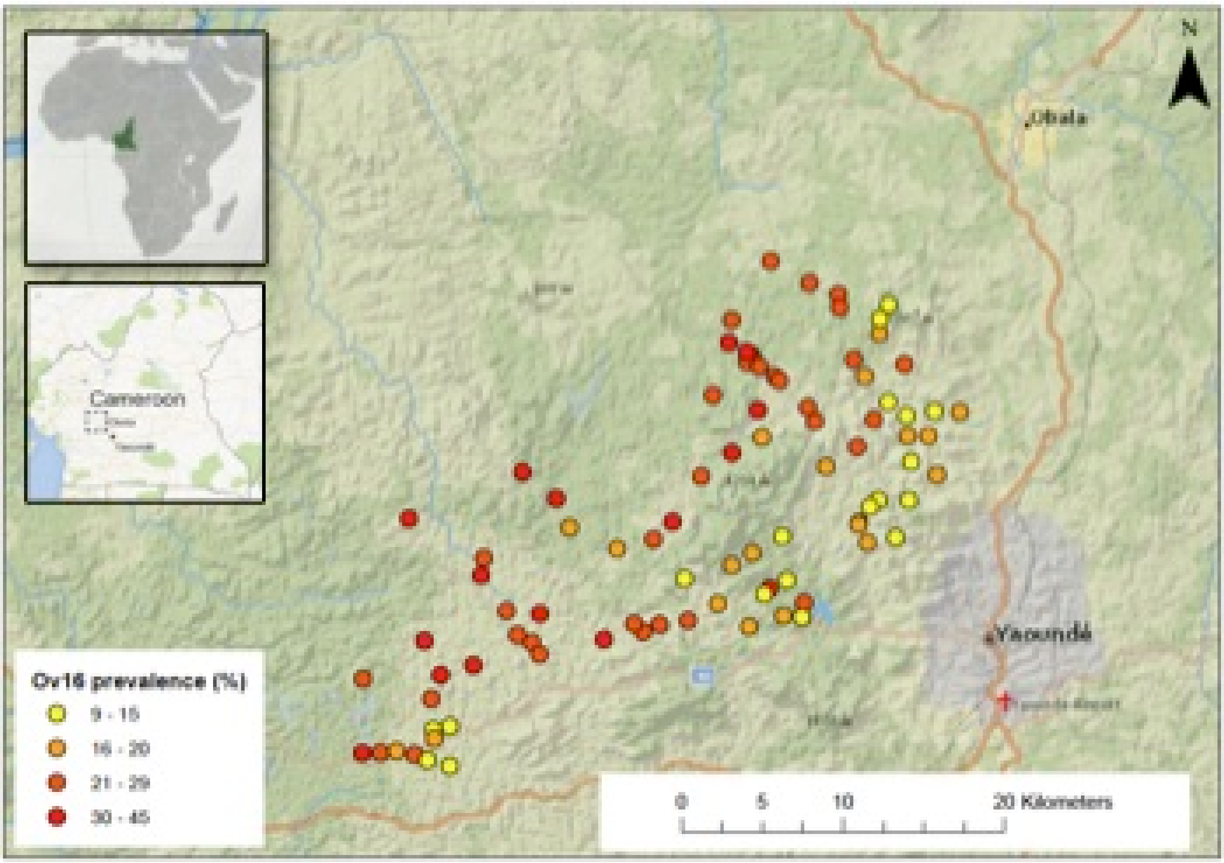

Figure S3. Timeline of the Test and not Treat strategy during year 2015

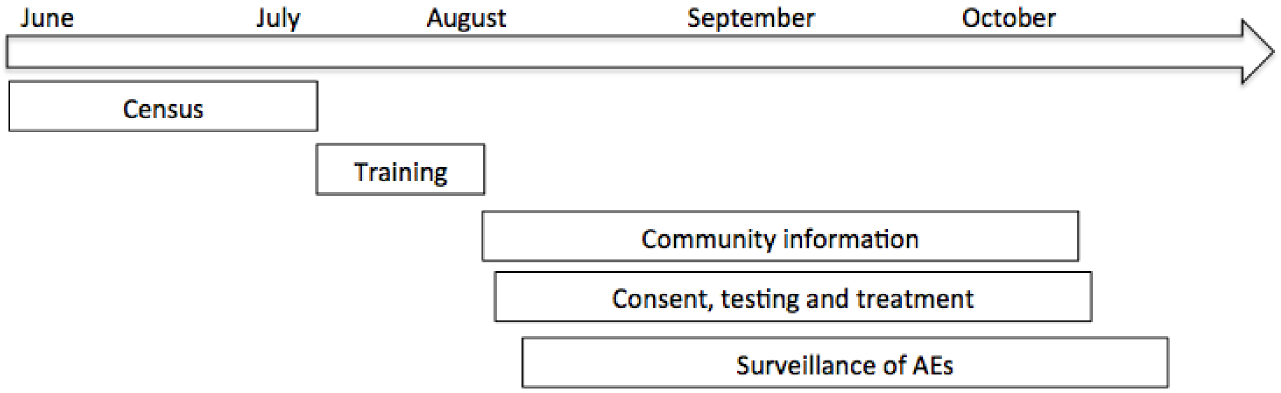

Figure S4. Individual card given to all participants

Date : ___________________	Village : ______________
N° TNT- ___________	N° ménage ________
Nom : ___________________	_________________
Prénom : _____________	Age ______ ans
Résultat LoaScope	_____________ mf/mL
Nb cp de Mectizan reçus	__________
Signature du participant	
Une équipe de surveillance passera 1, 2, 3, et 6 jours après le traitement dans votre village	En cas de réaction au traitement, revenez avec votre carte individuelle
Tél. de l'équipe:	**696 50 51 85**

Figure S5. Standardized form to record all adverse events following ivermectin treatment

« Test and treat » - Effets secondaires Date du jour : _____/______/2015

Première visite ☐ Visite de suivi ☐ Effectuée par (initiales) : ___________

Date du traitement par Mectizan _____/_______/ 2015 Village ____________________

N° TNT-____________ Numéro de ménage : _______ LoaScope J0:______________

Nom et prénom ____ Sexe ___ Age ____(ans)

Patient vu au point de consultation ☐ au domicile ☐ alité ☐

Interrogatoire : patient ☐ entourage ☐

Plaintes spontanées (noter heure de début, écrire au verso si nécessaire: ________________________

____

____

Questionnaire (entourer chaque réponse, ajouter + après si marqué)

Asthénie: Oui / Non Anorexie: Oui / Non Céphalées: Oui / Non Lombalgies: Oui / Non

Autres arthralgies: Oui / Non Myalgies: Oui / Non Prurit: Oui / Non

Troubles visuels: Oui / Non si oui, préciser ____

Troubles auditifs, vertiges: Oui / Non si oui, préciser ____

Antécédents médicaux: Oui / Non si oui, préciser ____

Carnet vu : Oui / Non

Traitement antérieur par IVM: Oui / Non / NSP si oui, date(s) :___________________________

Examen systématique

Rash: Oui / Non Œdèmes: Oui / Non Adénites: Oui / Non (préciser localisations au verso)

Hémorragie conjonctive palpébrale: Oui / Non, Précisions:___________________ Photo faite: Oui / Non

Difficultés à marcher: Oui / Non si oui, par: Douleur / Fatigue

Difficultés à se lever d'une chaise: Oui / Non si oui, préciser cause ____________________________

Mutisme: Oui / Non Obnubilation: Oui / Non Agitation: Oui / Non

Troubles de la conscience: Oui / Non si oui, Glasgow mesuré : Oui / Non Résultat: _____/15

Incontinence urinaire: Oui / Non autres troubles neuro : Oui / Non (si oui, préciser au verso)

Autres troubles (digestifs, etc.): Oui / Non si oui, préciser: __________________________________

Karnofsky (%): ____ Température: ____°C TA couché: ____/____ TA debout/assis: ____/____

Examens supplémentaires nécessaires (T° ≥ 38/5° ou indice Karnofsky ≤70%): Oui / Non

Si oui, remplir une fiche « Examens supplémentaires »

Traitement en cours: paracétamol / ibuprofène / chlorpheniramine / multivit. / quinine / SRO / artemether / amoxicilline / doxycycline / sulfate ferreux / hydroxyde d'Alu / métronidazole

Traitement donné ce jour: paracétamol / ibuprofène / chlorpheniramine / multivit. / quinine / SRO / artemether / amoxicilline / doxycycline / sulfate ferreux / hydroxyde d'Alu / métronidazole

Hospitalisation: Oui / Non Si oui, préciser :____

A remplir par l'opérateur de saisie: Informations au verso vérifiées ☐ Fiche saisie ☐

Figure S6. Number of excluded individuals in each village, classified by cause of exclusion (high *Loa* microfilaremia or other cause)

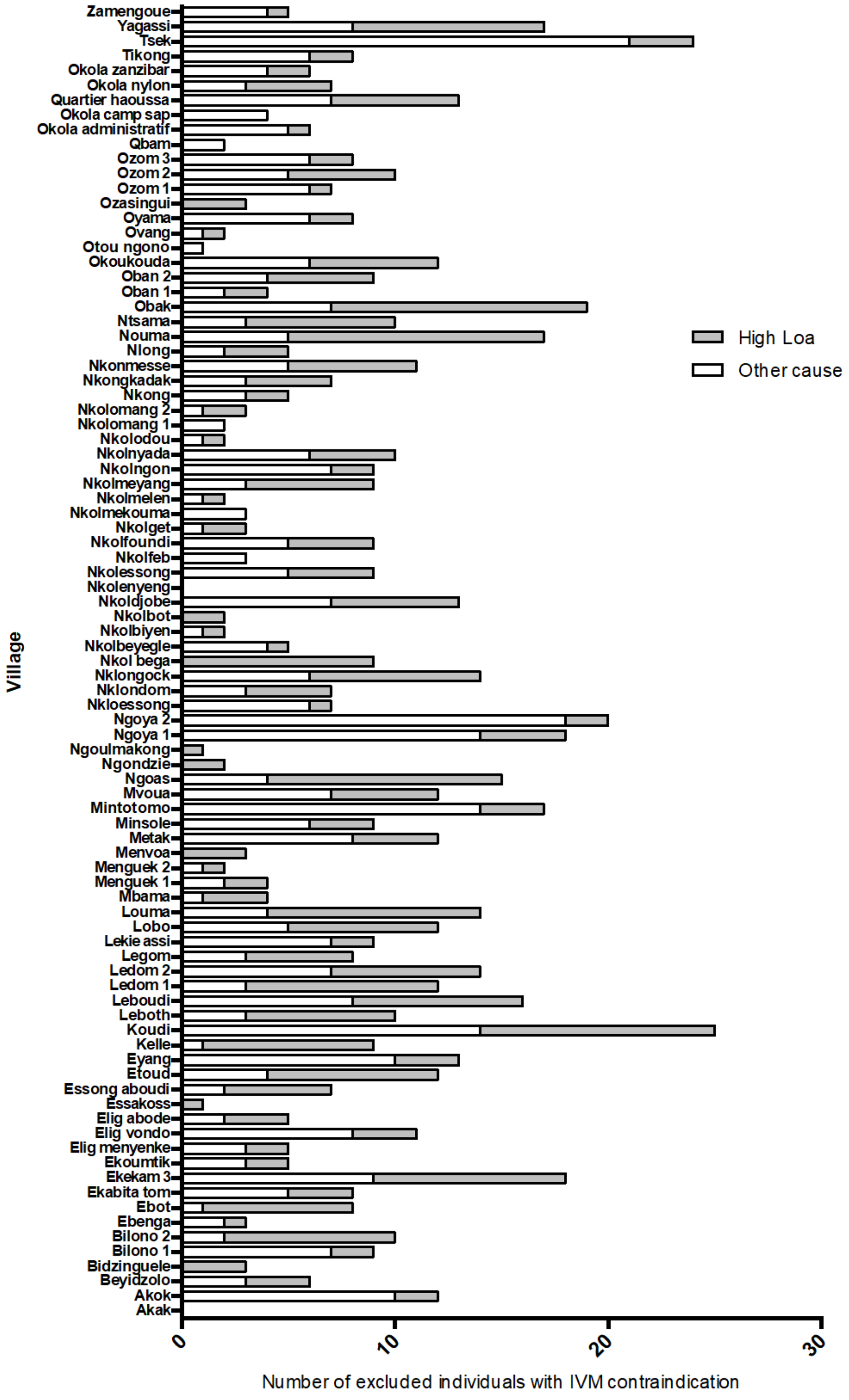

Movie S1. Link to a movie of the process from introduction of the capillary to the result.

https://www.dropbox.com/s/mctgg4i9ibz3768/LoaScope%20process.MP4?dl=0

Supplementary Methods: announcement of the TaNT campaign to the population

During a house to house census, the objectives and study design were explained informally to the head of each household, specifically highlighting the benefits of ivermectin treatment at the individual level (effect on filarial and intestinal worms, clinical improvement) and at the community level (impact on transmission) along with the potential risks of ivermectin-based MDA. During the week prior to the TaNT campaign, village authorities provided the population with logistical information, and flyers (available on demand) explaining the TaNT strategy were distributed to all households.
